# Comment on “Risk factors associated with the use of red blood cells in elective cardiac surgeries”

**DOI:** 10.1016/j.htct.2025.106236

**Published:** 2026-01-10

**Authors:** Hinpetch Daungsupawong, Viroj Wiwanitkit

**Affiliations:** a Phonhong, Lao People's Democratic Republic; b Department of Research Analytics, Saveetha Dental College and Hospitals, Saveetha Institute of Medical and Technical Sciences Saveetha University, Chennai, India

Dear Editor,

We herein discuss the publication on “Risk factors associated with the use of red blood cells in elective cardiac surgeries: A patient blood management (PBM) view”. [1] This article investigates the risk variables linked with the use of packed red blood cells (PRBC) in patients undergoing elective heart surgery in Brazil.

While stepwise logistic regression results show that female gender, poor hematocrit level, diabetes, and extracorporeal circulation duration of more than 90 min are significant risk variables, this analysis is statistically limited. As this is a retrospective study, it is susceptible to bias from data collection or sample selection. Furthermore, stepwise logistic regression may result in overfitting and variable selection based on the specific data set, restricting its applicability to other hospitals or populations.

Furthermore, underlying heart disease severity, changes in surgeon treatment, surgical technique, or anticoagulant use can all have an impact on the accuracy of the outcomes as these variables may not be recorded or controlled in the analysis. Furthermore, the identification of female gender as a high-risk factor could be explained by variations in body weight or total blood volume, however the paper did not account for these variables in its analysis. This may confound the relationship.

New interpretations may concentrate on synthesizing findings for PBM guidelines. Preoperative hematocrit assessment and improvement of anemia, diabetes management, and surgical planning to limit cardiopulmonary bypass time can all help to lessen the requirement for PRBC. Furthermore, including risk prediction models that take into account characteristics such as gender, body weight, disease severity, and laboratory results might improve accuracy and simplify effective blood use planning.

To broaden the topic, consider the following research questions:

1) Can this retrospective study be transformed into a prospective or multicenter study to corroborate the relationship?
2) How much can enhancing PBM with preoperative optimization techniques reduce PRBC use and clinical outcomes?
3) How do individual characteristics, such as body mass index and female gender, affect overall blood volume?
4) Can multivariate machine learning algorithms accurately predict the requirement for PRBC in cardiac surgery patients?

These questions may lead to applicable research as well as safe, resource-saving strategies in Brazil's health-care system.

## Data availability statement

there Is no new data generated

## Funding statement

there is no funding

## Ethics of approval statement

not applicable

## Patient consent statement

not applicable

## Permission to reproduce material from other sources

not applicable

## Clinical trial registration

not applicable

## Author contributions

HP 50 % ideas, writing, analyzing, approval

VW 50 % ideas, supervision, approval

## AI declaration

the authors use computation tool in language checking and editing.

## Conflicts of interest

the authors declare no conflict of interest
